# P03-025 – Differential diagnosis of autoimmune disorders

**DOI:** 10.1186/1546-0096-11-S1-A223

**Published:** 2013-11-08

**Authors:** A Kozlova, N Zinovieva, J Bustamante, S Boisson-Dupuis, J-L Casanova, A Shcherbina

**Affiliations:** 1 Immunology, Federal Research and Clinical Center for Pediatric Hematology, Oncology and Immunology, Moscow, Russian Federation; 2 Immunology, Children’s Clinical Hospital #9, Moscow, Russian Federation; 3 Laboratory of Human Genetics of Infectious Diseases, Institut National de la Santé et de la Recherche Médicale, Paris, France; 4 St. Giles Laboratory of Human Genetics of Infectious Diseases, The Rockefeller University, New York, USA

## Introduction

Mendelian susceptibility to mycobacterial disease (MSMD) is a rare form of primary immunodeficiencies characterized by predisposition for poorly virulent infection agents, primarily non-tuberculous mycobacteria and Salmonella. It has been shown that molecular basis of these diseases is mutations in at least 7 genes in the IL12-dependent IFN-g axis including *IFNGR1*, *IFNGR2*, *IL-12/IL23RB1*, *IL-12B*, *IRF8*, *NEMO*, *CYBB* and *STAT1.* There are about 140 patients with IL-12Rb1/IL-23Rb1 deficiency reported up to date, who manifest with various nonspecific inflammatory features due to chronic BCG infection and salmonellosis.

## Case report

Here we report two patients ages 4 and 8 who were referred to us with preliminary diagnosis of autoimmune disorder and very similar symptoms of enlarged lymph nodes of several groups, recurrent fever and vasculitic rash on extremities. In the second patient the skin rash was considered a manifestation of vasculitis and, as a result, she was treated with glucocorticoids and azathioprine for more than 5 years with short interruptions and minimal effect. Laboratory tests in both patients showed: anemia 75-100 g/l, increased inflammatory activity (CRP, ESR), hyper-gammaglobylinemia (IgG 27-39.4 g/l; IgA 0.65 – 9.17; IgM 1.5 - 3.11 g/l). Both patients had very high levels of rheumatoid factor (with no arthritis), low levels of C4 complement. ANF levels were normal.

## Discussion

It was known that both patients had regional BCG infection following vaccination in early infancy, that required massive anti-mycobacterial therapy. Salmonellosis complicated by pneumonia was diagnosed at 2 and 6 years of age in the 1^st^ and 2^nd^ patient, respectively.

Upon admission to our Center both patients’ blood cultures were positive for *Salmonella D. enteritidis*. The extensive anti-bacterial therapy and IFN-g s.c. without any antiinflamatory treatment provided good control of the disease in both girls.

The diagnosis of IL12Rb1 deficiency in these patients was based on *in vitro* findings of nulle expression of CD212 (IL-12Rb1) on PHA-stimulates T cell blasts, decreased *in vitro* production of INFg by PHA activated PBMC and was genetically confirmed.

## Conclusion

In accessing patients with periodic fever and autoimmune-like features it is important to keep in mind a group of rare primary immunodeficiencies, affecting IL12\IFN gamma axis.

## Disclosure of interest

None declared

